# Application of AI on cholangiocarcinoma

**DOI:** 10.3389/fonc.2024.1324222

**Published:** 2024-01-29

**Authors:** Jianhao Huang, Xuesong Bai, Yanyu Qiu, Xiaodong He

**Affiliations:** Department of General Surgery, State Key Laboratory of Complex Severe and Rare Diseases, Peking Union Medical College Hospital, Peking Union Medical College and Chinese Academy of Medical Sciences, Beijing, China

**Keywords:** cholangiocarcinoma (CCA), machine learning (ML), artificial intelligence, prognosis, diagnosis

## Abstract

Cholangiocarcinoma, classified as intrahepatic, perihilar, and extrahepatic, is considered a deadly malignancy of the hepatobiliary system. Most cases of cholangiocarcinoma are asymptomatic. Therefore, early detection of cholangiocarcinoma is significant but still challenging. The routine screening of a tumor lacks specificity and accuracy. With the application of AI, high-risk patients can be easily found by analyzing their clinical characteristics, serum biomarkers, and medical images. Moreover, AI can be used to predict the prognosis including recurrence risk and metastasis. Although they have some limitations, AI algorithms will still significantly improve many aspects of cholangiocarcinoma in the medical field with the development of computing power and technology.

## Introduction

Cholangiocarcinoma (CCA) is an advanced and lethal malignancy with a rare incidence of the disease (1). It arises from the epithelial cells of the biliary ducts. According to the original anatomical site, cholangiocarcinoma is divided into intrahepatic, perihilar, or extrahepatic (2). The annual incidence is approximately 1.26 cases of cholangiocarcinoma per 100,000 people, and two-thirds of cases are intrahepatic type in the United States (3). The signs and symptoms associated with cholangiocarcinoma are non-specific. The location of the lesion is suggested by the patient’s clinical presentation and the initial radiographic findings (4). Extrahepatic cholangiocarcinoma becomes symptomatic when the biliary drainage system is obstructed, including jaundice, pruritus, and color change of the stools and urine. Intrahepatic cholangiocarcinoma is usually asymptomatic and less possibly presents jaundice (5). Tumor markers, including CEA, CA 19-9, and even the combined parameters of CA 19-9 and CEA, do not have enough specificity and accuracy to detect early-stage cholangiocarcinoma (6–8). Transabdominal ultrasound, as the first appropriate imaging study for patients with jaundice, has a high sensitivity to detect the main reason for the biliary tract dilation and evaluate potential vascular involvement. However, early-stage distal extrahepatic cancers may not be detected due to the limited visualization of the distal common bile duct, especially if the small lesion does not make the bile duct become visibly dilated (9, 10). CT, MRI, and PET/CT are not recommended for asymptomatic populations as routine examinations due to the low incidence of cholangiocarcinoma and the financial cost involved (2, 11–13). ERCP and other invasive procedures including percutaneous cholangiography, brush cytology, and endoscopic ultrasound are also not recommended (14, 15). Only a minority of patients present with early-stage disease and are considered candidates for resection, which is the only possible cure (1, 6, 16). Consequently, early diagnosis of cholangiocarcinoma is still challenging nowadays.

Artificial intelligence (AI) was first proposed by John McCarthy et al. in 1956, where it was widely considered as “thinking machines.” AI is defined as investigating and developing a digital computer or computer-controlled machine to simulate and perform the intellectual process characteristic of humans (17). AI has shown great success in a wide variety of medical studies, including radiology (18, 19), pathology (20), gastroenterology (21), and ophthalmology (22). AI has a great advantage in analyzing vast amounts of data and identifying patterns and trends of disease, such as histologic, cytologic, serum marker, and radiologic methods. In the early diagnosis of cholangiocarcinoma, AI can analyze the input histologic, cytologic, or radiologic images to extract features that are specific for cholangiocarcinoma but not fully recognized by humans, with the type of dimensionality reduction (23). Decision tree algorithms can be trained with serum markers to determine the better cutoff value and workflow for the diagnosis of cholangiocarcinoma. Moreover, AI efficiently automates the repetitive jobs for physicians, achieving more rapid identification of the suspected nodule (17, 24). With the continuous improvement of the computing power of computers, AI is the driving force in the development of new treatments and technologies in the medical field.

However, there are still some disadvantages to the application of AI in the medical field, particularly in building user trust in AI systems (25). Interpretability means that the cause and effect can be determined (26). Nowadays, some AI algorithms function as uninterpretable “black boxes” and are difficult to explain how cholangiocarcinoma is being predicted by the input information. Artificial intelligence itself cannot be the subject of a legal relationship so a framework for determining the responsibilities and legal liability for AI is also needed (27). Furthermore, medical data from different institutions may be varied in quality and complicated in feature labeling (28). It also raises our concerns about data privacy during the application of AI (29). Most AI applications currently approved by the FDA are studied in retrospective studies, while few prospective studies investigated the clinical application of AI algorithms (30).

In this review, “artificial intelligence,” “machine learning,” and “cholangiocarcinoma” were used as the keywords. Relevant literature published until September 2023 in PubMed, Embase, Web of Science, and other databases were reviewed. We highlighted the AI function in serum biomarkers, radiologic analysis, and pathological examination in the tumor diagnosis process. In prognosis prediction, recurrence risk, metastasis, and overall survival of cholangiocarcinoma are investigated by AI. Finally, we discussed the future challenges for cholangiocarcinoma management.

## Machine learning

Machine learning (ML) is a branch of artificial intelligence. By making use of data and algorithms, it simulates the way how humans think and execute (31). Machine learning can handle large volumes of data and discover potential trends, especially in radiology. The number of images often exceeds the processing capacity of radiologists, while it can be rapidly studied by ML to localize the cholangiocarcinoma for radiologists (32). Also, it is widely used in the three-dimensional reconstruction of the biliary system from the images so that the area of malignancy can be automatically delineated for surgery and radiotherapy (33, 34).

The performance of an ML model is usually measured by the AUROC, which determines the tradeoff value between sensitivity and specificity (35). Other parameters, including accuracy, C-index, and PPV, are also commonly used to evaluate the predictive result of ML. The higher the values of the parameters are, the more accurate the prediction of cholangiocarcinoma can be achieved.

## Supervised learning

**Figure 1:**
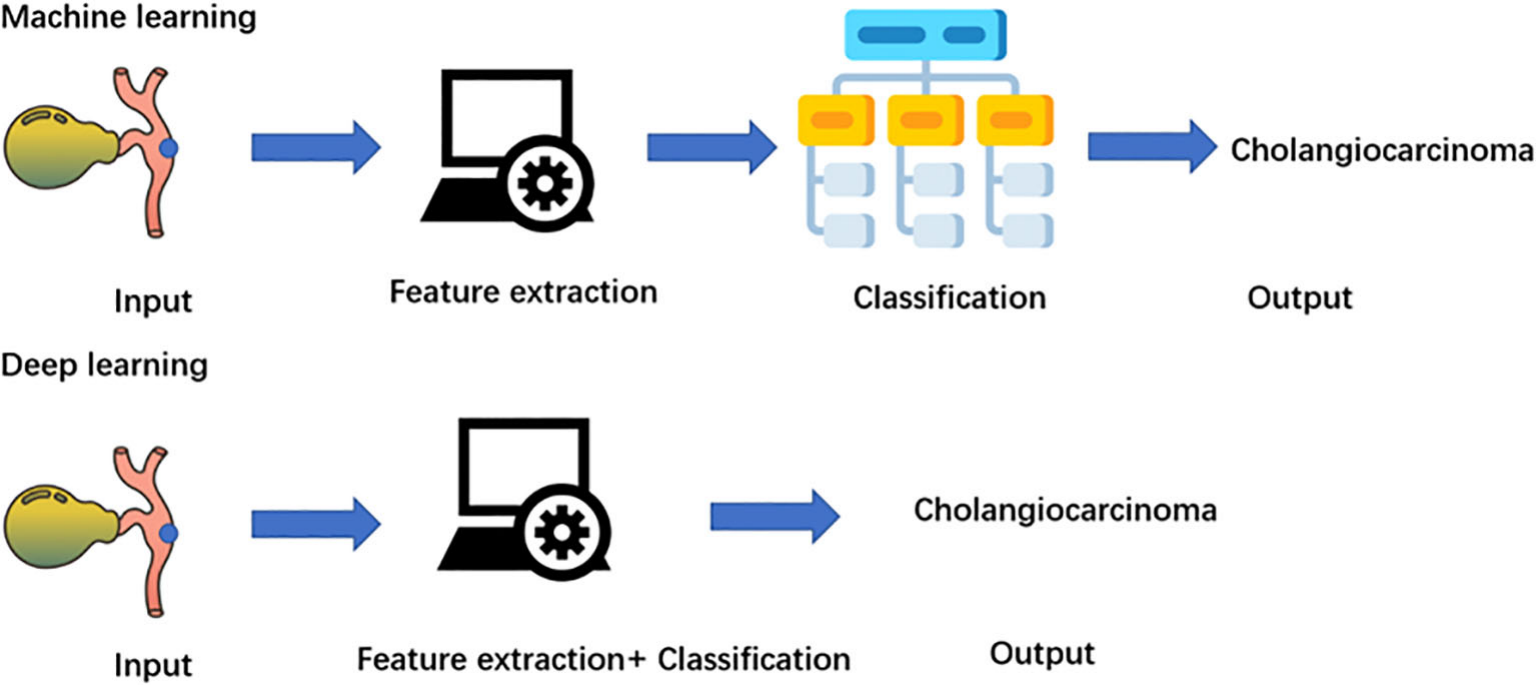
Machine learning can be classified as supervised, unsupervised, semi-supervised and reinforcement learning. All data is labeled In supervised learning, while unsupervised learning is unlabeled. Small amount of labeled data fed semi-supervised learning, and a large amount of unlabeled data can be handled. Reinforcement learning interact with rewards or punishments from the environment.

Supervised learning is a training algorithm that uses labeled features to classify data and predict outcomes accurately (36). For cholangiocarcinoma, clinical physicians labeled the specific site of malignancy in radiology or abnormal serum biomarkers for malignancy. Then, the labeled data are fed into the model, and the cross-validation process is performed to adjust the weights appropriately for diagnosis. In this review, most of the models are established by supervised learning, typically in the differential diagnosis part of cholangiocarcinoma with clinical features and radiomic features (**Figure 1**).

## Unsupervised learning

Without the need for human intervention for images of cholangiocarcinoma, unsupervised learning analyzes unlabeled data and discovers similarities and differences (37). Potential patterns or data groupings can be identified by unsupervised learning. Also, it can be used to map the original high-dimensional data to achieve dimensionality reduction, typically in radiomic studies for cholangiocarcinoma. PCA, SVD, and k-means clustering are some common algorithms.

## Semisupervised learning

In semisupervised learning, clinical physicians label smaller data for cholangiocarcinoma and feed the algorithms to perform learning tasks on a bigger unlabeled database for the diagnosis of cholangiocarcinoma (38). Semisupervised learning overcomes the disadvantage of supervised learning, which requires large enough labeled data. Moreover, using labeled data can prevent models from learning false correlations to improve the accuracy of the model.

## Reinforcement learning

Reinforcement machine learning is an ML model that is not trained by sample data (39). This model learns by using trial and error and simulates the process to maximize the cumulative reward. For cholangiocarcinoma, the goal-oriented algorithm accumulates the successful treatment outcomes for cholangiocarcinoma to develop the best recommendation. Therefore, specific patients who may benefit from the surgery can be identified.

## Deep learning

**Figure 2:**
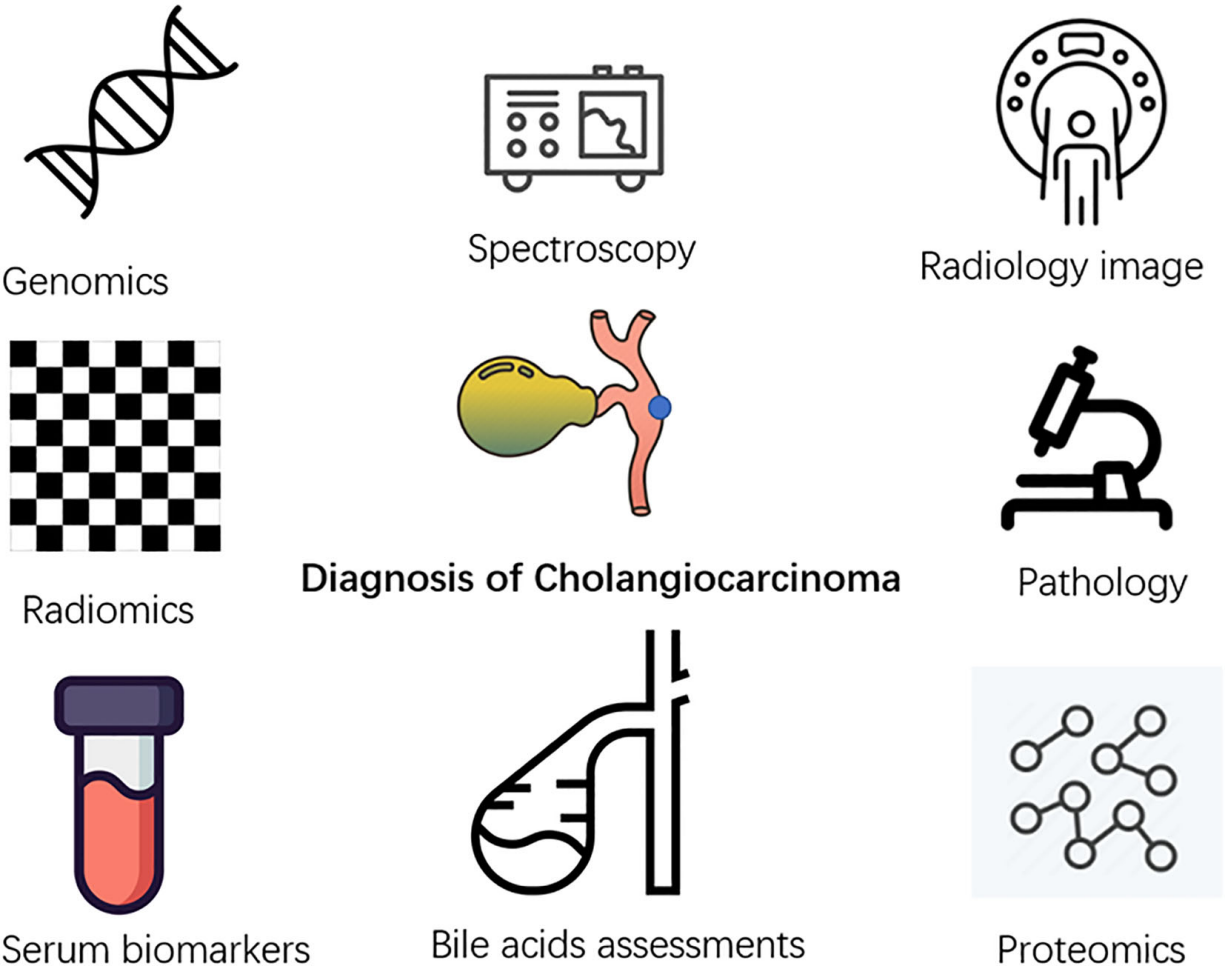
Application of artificial intelligence in multiple related fields of diagnosis of cholangiocarcinoma.

Deep learning is a branch of machine learning that focuses on deep artificial neural networks (40). Compared with machine learning algorithms, deep learning eliminates some of the data preprocessing and ingests the unstructured raw data (**Figure 2**). The features for the classification of cholangiocarcinoma are automatically extracted and analyzed to determine the most important features to distinguish cholangiocarcinoma, such as the biliary tract infiltration and the tumor size. Furthermore, it adjusts and fits itself for accuracy through the processes of gradient descent and backpropagation, especially when handling high-dimensional data. Deep learning algorithms, such as CNN and RNN, have been widely used in radiomics for cholangiocarcinoma and have achieved great success in image classification in recent years (41–43).

## AI and diagnosis

**Figure 3:**
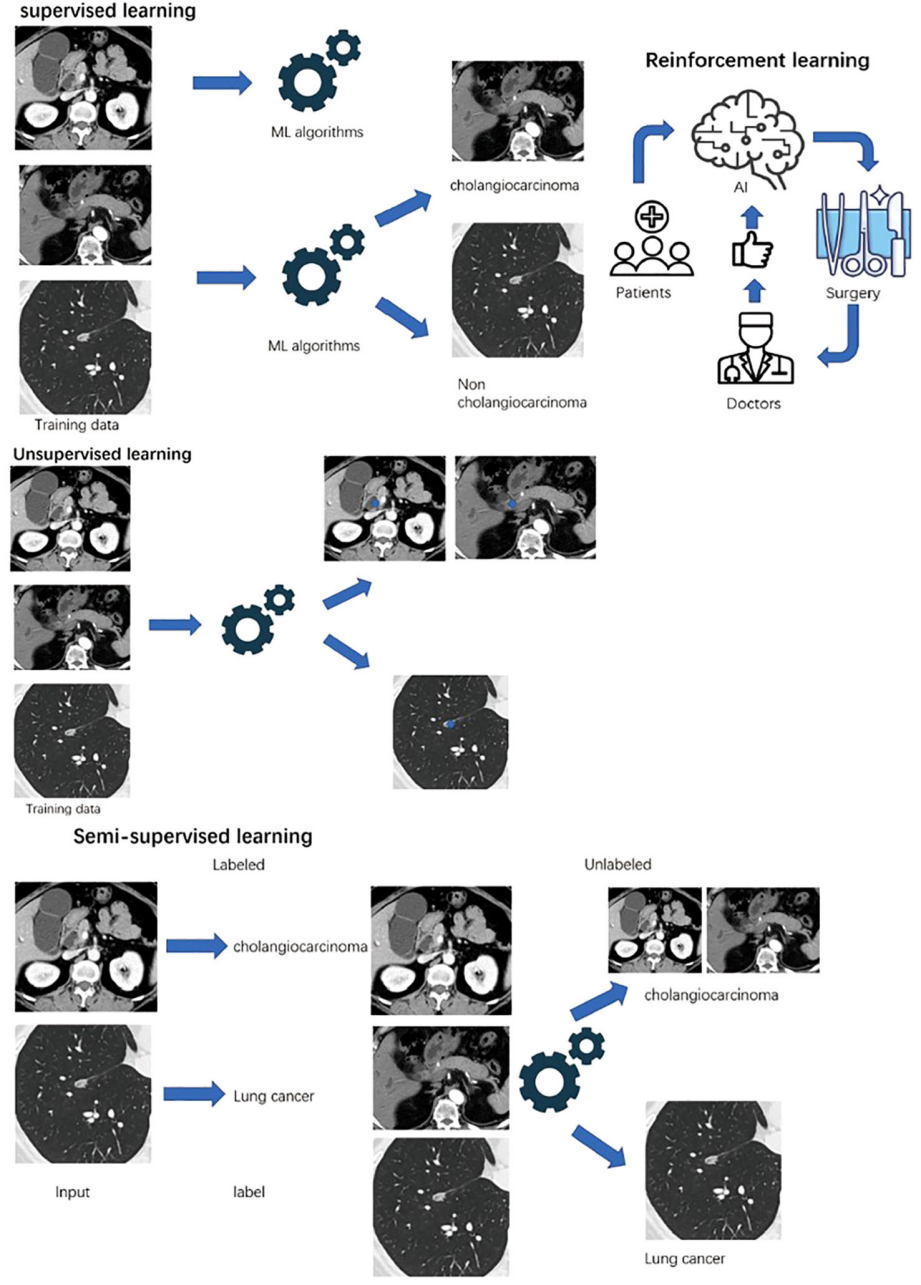
Diagram of machine learning and deep learning process. Machine learning needs four steps: input, feature extraction, classification, and output. Deep learning is a subgroup of a machine learning algorithm, which can automatically extract labels.

Cholangiocarcinoma lacks accurate early diagnostic methods, which are important for patients at high risk of suffering from primary sclerosing cholangitis, polycystic liver disease, and chronic hepatolithiasis (44). Many researchers currently focus on applying AI to the early diagnosis of cholangiocarcinoma using different clinical information of the patients (**Figure 3**).

**Table 1** summarized the application of AI in diagnosis of cholangiocarcinoma. Spectroscopy is considered a useful method for identifying subtle changes associated with cancer development in biological samples (45, 46). Making use of patients’ serum samples, Su et al. (47) applied Raman spectroscopy to identify typical biomolecular components in CCA and achieved good diagnostic accuracy combined with the SVM algorithm. Giordano et al. (48) also applied mass spectrometry with SVM and RF to permit cholangiocarcinoma identification, with an accuracy of 99.0%, sensitivity of 98%, and specificity of 100%. Furthermore, sera from CCA patients were detected by Chatchawal et al. to find out specific molecular vibrations of molecules and gain cancer-specific biomarkers using attenuated total reflectance-Fourier transform infrared (ATR-FTIR) spectroscopy (49). SVM, RF, and NN models were established. The variations of collagen molecules, phosphate groups, lipid ester carbonyls, and polysaccharides were found in CCA patients. Specific lipid metabolism in HCC and abnormal extracellular pathways in CCA were found by mass spectrometry (50). RF was used to select the features and establish the model with an area under the curve (AUC) value of 0.92 and an accuracy of 90%. To differentiate adenocarcinomas of the pancreas and biliary tree, Bollwein et al. (51) investigated the mass spectrometric peptide sequences of histone and collagen with GB, SVM, and KNN algorithms, achieving an AUC of 0.96 and accuracy of 0.91. Serum lipid and peptide detection was conducted by nano-assisted laser desorption ionization mass spectrometry to discriminate between benign biliary diseases and cholangiocarcinoma (52). Three algorithms (ANN, LASSO, SVM) were used to investigate the lipidomics panel, peptidomics panel, and multiomics panel, achieving sensitivity and specificity of 96.5% and 96.4% in the diagnosis.

**Table 1:** Application of AI in the diagnosis of cholangiocarcinoma.

Reference	Sample size	Data source	Algorithms	Aim	Best result
Tang et al.	100 cases	1,200 radiomics features extracted from axial T1WI, T2WI, DWI, and ADC	Bagging classifier	Differentiate the degree of ECC	AUC 0.90, accuracy 0.85, sensitivity 0.75, and specificity 0.88
Tang et al.	100 cases	1,200 radiomics features extracted from axial T1WI, T2WI, DWI, and ADC	Extreme gradient boosting classifier	Predict lymph node metastasis of ECC	AUC 0.98, accuracy 0.90, sensitivity 0.75, and specificity 0.94
Shen et al.	2,269 cases	Clinical features	LASSO regression and random forest	Predict lymph node metastasis of ICC	Accuracy 82.6%, AUC 0.867
Xu et al.	129 cases	CE-CT	distance correlation (DC)_LDA and RF_LDA	Differentiate ICC and hepatic lymphoma	Accuracy 96.2%, AUC 0.997
Ren et al.	226 cases	Ultrasound	SVM	Differentiate between HCC and ICC	AUC 0.936, sensitivity 0.900, specificity 0.857, and accuracy 0.868
Liu et al.	85 cases	MRI	SVM	Differentiate between combined hepatocellular cholangiocarcinoma	AUC 0.77
Hu et al.	24 cases	Multiphasic MRI	Tree-based pipeline optimization tool	Differentiate between HCC and ICC	Accuracy 73%–75%, sensitivity 65%–75%, specificity 75%–79%
Liu et al.	100 cases	MRI and clinical features	LASSO, Gaussian process regression, KNN, LR, partial least squares-discriminant analysis, quadratic discriminant analysis, RF, SGD, SVM, and XGBoost	Predict VEGF expression and MVD of ECC	AUC 0.912
Liao et al.	166 differentially expressed genes	Gene Expression Omnibus (GEO) database	RF and ANN	Diagnosis of cholangiocarcinoma	AUC 0.980
Giordano et al.	96 cases	Probe electrospray ionization mass spectrometry	SVM and RF	Diagnosis of cholangiocarcinoma	Accuracy 99.0%, sensitivity 98%, specificity 100%
Liu et al.	112 cases	MRI	MFF, SRB, and CBAM	Differentiate between HCC and ICC	AUC 0.9680, accuracy 92.26%, sensitivity 86.21%, and specificity 94.70%
Chen et al.	134 cases	Gd‐EOB‐DTPA-enhanced MRI	SelectKBest, LASSO, LR, RF, SGD, SVM	Differentiate between atypical ICC and poorly differentiated HCC	AUC = 0.90
Urman et al.	129 cases	Mass spectrometry and nuclear magnetic resonance spectroscopy	NN	Differentiate between benign biliary strictures and cholangiocarcinoma	AUC 0.984, sensitivity 94.1%, and specificity 92.3%
Dragomir et al.	760 cases	genome-wide DNA methylation data	NN, SVM, and RF	Differentiate between ICC and hepatic metastases of pancreatic ductal adenocarcinoma	Accuracy 100%
Mahmoudi et al.	94 cases	CT radiomics features and clinical features	t-SNE, LASSO, LR, ADB, SGB, and RF	Differentiate between HCC and ICC	AUC = 0.82, sensitivity 0.733, and specificity 0.857
Yi et al.	107 cases	Mass spectrometry	RF	Differentiate between HCC and ICC	AUC 0.92 and accuracy 90%
Huang et al.	494 cases	CT	LR, LASSO, SVM and RF	Differentiate between HCC and ICC	AUC 0.987, accuracy 0.939
Wang et al.	179 cases	CT radiomics and clinical features	SVM	Predict lymph node staging for hilar cholangiocarcinoma	AUC 0.870
Piansaddhayanon et al.	3 cases	Microscopy image of organoid-derived cells	NN	Detect the circulating tumor cell in the blood sample	AUC 0.78
Silverstri et al.	543 cases	RNA-seq and microarray platforms	KNN, SVM, and fast unified RF	Diagnosis of cholangiocarcinoma	AUC 0.99
Watcharatanyatip et al.	63 cases	Translational proteomic	SVM	Diagnosis of cholangiocarcinoma	AUC 0.96
Murugesan et al.	6,518 cases	Genomic profiles	RF	Diagnosis of combined hepatocellular cholangiocarcinoma	Sensitivity 85.9%, specificity 93.4%, and accuracy 91%
Kiani et al.	80 cases	hematoxylin and eosin-stained whole-slide images	CNN	Pathologically differentiate between HCC and cholangiocarcinoma	Accuracy 0.885
Peng et al.	589 cases	Ultrasound-based radiomics	Extremely randomized trees, RF, LASSO, naive Bayes, SVM	Differentiate between infected focal liver lesions and ICC	AUC 0.949
Wang et al.	196 cases	MRI radiomics	LASSO, SVM	Diagnosis of ICC	AUC 0.91, sensitivity 0.88, specificity 0.89, accuracy 0.89
Xu et al.	211 cases	CT radiomics features	LASSO, SVM	Differentiate between HCC and ICC	AUC 0.855
Huang et al.	149 cases	CT/MR image and serum biomarker	RF	Predict ICC lymph node metastasis	AUC 0.758, C-index 0.837, sensitivity 0.82, specificity 0.90, PPV 0.93, NPV 0.75, false positive 0.41, false negative 0.28, accuracy 0.85
Gao et al.	644 cases	Proteomics data	LASSO, RF, t-SNE	Diagnosis of cholangiocarcinoma	AUC of 0.947, sensitivity of 90.3%, specificity of 84.9%, accuracy of 87.0%
Ponnoprat et al.	257 cases	Multiphase CT	NN, KNN, DT, RF, MLP, and SVM	Differentiate between HCC and ICC	Accuracy 88.19%
Ali Shah et al.	516 cases	Gene sequence	LSTM and GRU	Diagnosis of cholangiocarcinoma	Accuracy 99%, sensitivity 100%, specificity 98%
Bollwein et al.	82 cases	mass spectrometric peptide features	GB, SVM, and KNN	Differentiate adenocarcinomas between the pancreas and biliary tree	AUC 0.96, accuracy 0.91
Wa et al.	120 cases	conventional ultrasound and contrast-enhanced ultrasound	LASSO	Differentiate between hepatic alveolar echinococcosis and ICC	Sensitivity 82.5%, specificity of 86.4%, AUC 0.913
Chatchawal et al.	46 cases	Serum markers in attenuated total reflectance-Fourier transform infrared spectroscopy	SVM, RF, and NN	Diagnosis of cholangiocarcinoma	Sensitivity 80%–100%, specificity 83%–100%
Qu et al.	235 cases	Serum multiomics	ANN, SVM, LASSO	Accurate discrimination of benign biliary diseases and cholangiocarcinoma	Sensitivity 96.5%, specificity 96.4%
Peng et al.	668 cases	Ultrasound-based radiomics	LASSO	Diagnosis of cholangiocarcinoma	AUC 0.920
Swain et al.	51,927 cases	Single-cell transcriptome analysis	KNN, SNN, UMAP, and t-SNE	Diagnosis of cholangiocarcinoma	–
Wu et al.	2 GEO databases	Gene expression profiles	LASSO, SVM-RFE, RF	Diagnostic gene of intrahepatic cholangiocarcinoma	AUC 0.999
Sun et al.	880 scenes	Multidimensional hyperspectral cholangiocarcinoma images	CNN, RF	Diagnosis of cholangiocarcinoma	Accuracy 88.2%
Wang et al.	494 cases	Multiphasic MRI	CNN	Diagnosis of cholangiocarcinoma	Positive predictive value 83.3%
Midya et al.	814 cases	Portal venous phase CT	Inception v3 network	Diagnosis of cholangiocarcinoma	Accuracy 96%, sensitivity 94%
Starmans et al.	486 cases	T2-weighted MRI radiomics	SVM, RF, LR, linear and quadratic discriminant analysis, Gaussian naive Bayes, ADB, and XGBoost	Diagnosis of cholangiocarcinoma	AUC 0.78
Hamm et al.	494 cases	Multiphasic MRI	CNN	Diagnosis of cholangiocarcinoma	Accuracy 92%, sensitivity 92%, specificity 98%, AUC 0.992
Gao et al.	519 cases	MRI	CNN	Preoperative prediction of microvascular invasion in intrahepatic cholangiocarcinoma	AUC 0.888, accuracy 86.8%, sensitivity 85.7%, specificity 87.0%, positive predictive value 63.2%, negative predictive value 95.9%
Zhu et al.	138 cases	CE-CT	SVM	IDH mutation status of ICC	Accuracy 0.863, sensitivity 0.727, specificity 0.885, AUC 0.813
Wolff et al.	11 cases	Optical coherence tomography	CNN	Differentiate between ICC and liver parenchyma	Sensitivity 0.94, specificity 0.93
Xu et al.	106 cases	T1-weighted contrast-enhanced MR images	SVM, mRMR	Predict lymph node status of ICC	AUC 0.870
Nakai et al.	617 cases	CT and tumor marker	CNN	Differentiate between HCC and ICC	Accuracy 0.61, specificity 0.68
Guo et al.	342 cases	Dynamic contrast-enhanced MRI radiomics and clinical features	LASSO	Differentiate between combined hepatocellular-cholangiocarcinoma and HCC	AUC 0.863, specificity 0.918, and sensitivity 0.738
Matake et al.	120 cases	Dual-phase CE-CT	ANN	Diagnosis of cholangiocarcinoma	AUC 0.961
Su et al.	38 cases	Serum Raman spectroscopy	SVM	Diagnosis of cholangiocarcinoma	sensitivity 94.55%, specificity 98.18%
Negrini et al.	112 cases	Plasma bile acid profiles	LR, KNN, naive Bayes, RBF SVM, RF, XGBoost	Screening of Cholangiocarcinoma	Area under the curve (AUC) 0.95, sensitivity 0.79, specificity 1.00, positive predictive value 1.00, negative predictive value 0.73, accuracy 86.4%
Tsilimigras et al.	826 patients	Tumor size and median CA 19-9 and NLR	Classification tree	differentiate between HCC and ICC	*κ* = 0.93
Jiang et al.	127 cases	18F-FDG PET/CT radiomic features	Sequential forward floating	Differentiate between HCC and ICC	AUC 0.90, accuracy 0.77, sensitivity 0.75, specificity 0.80

CE-CT, contrast-enhanced computed tomography; MRI, magnetic resonance imaging; T1WI, T1-weighted imaging; T2WI, T2-weighted imaging; DWI, diffusion-weighted imaging; ADC, apparent diffusion coefficient; VEGF, vascular endothelial growth factor; MVD, microvessel density; AP, arterial phase; PVP, portal venous phase; 18F-FDG PET/CT, 18F-fluorodeoxyglucose positron emission tomography/computed tomography; CA 19-9, carbohydrate antigen 19-9; NLR, neutrophil-to-lymphocyte ratio; SBRT, stereotactic body radiotherapy; DL, deep learning; LASSO, least absolute shrinkage and selection operator; RF, random forest; SVM, support vector machine; ANN, artificial neural network; MFF, multilayer feature fusion module; SRB, stationary residual block; CRAM, convolutional block attention module; LR, logistic regression; SGD, stochastic gradient descent; NN, neural network; t-SNE, t-distributed stochastic neighbor embedding; ADB, AdaBoost; SGB, stochastic gradient boosting; KNN, K-nearest neighbors; CNN, convolutional neural network; MLP, multilayer perception; DT, decision tree; LSTM, long short-term model; GRUs, gated recurrent units; SNN, shared nearest neighbor; UMAP, uniform manifold approximation and projection; XGBoost, extreme gradient boosting; mRMR, maximum relevance minimum redundancy; GBM, gradient boosting machine; CART, classification and regression tree; LDA, linear discriminant analysis; QDA, quadratic discriminant analysis; ICC, intrahepatic cholangiocarcinoma; ECC, extrahepatic cholangiocarcinoma; HCC, hepatocellular carcinoma; PHC, perihilar cholangiocarcinoma; AUC, area under the curve; NPV, negative predictive value; PPV, positive predictive value.

Bile acid assessments are potentially involved in the development and progression of CCA (53). Using plasma bile acid profiles, the performance of six ML algorithms was evaluated by Negrini et al. (54). Naive Bayes, using direct bilirubin concentration as the normalization process, achieved the best performance, with an AUC of 0.95, specificity of 1.00, PPV of 1.00, and accuracy of 86.4%, which was significantly better than the other five algorithms (LR, KNN, RBF SVM, RF, XGBoost). CLU protein was upregulated in bile proteomics in CCA, proven by immunoblotting analysis, qRT-PCR, and immunohistochemistry staining. Combined with serum CA 19-9, indirect bilirubin, GGT, TG, LDLC, and TBA levels, the seven-panel models with LASSO and RF methods had an AUC of 0.947, sensitivity of 90.3%, specificity of 84.9%, and accuracy of 87.0% (55). Urman et al. (56) further analyzed the metabolomics and proteomics of human bile with spectrometry. The NN algorithm was performed to extract useful metabolomic data. A total of 15 molecular features were identified with an AUC of 0.984, sensitivity of 94.1%, and specificity of 92.3% in differentiating CCA from benign biliary stenoses.

Differentially expressed genes are also available for CCA diagnosis (57). Human gene expression profiles of the ICC GEO database were investigated to screen out the differentially expressed genes with three algorithms (LASSO, SVM-RFE, RF). MMP14 was finally determined with high accuracy and an AUC of 0.999, which affects the infiltration of monocytes and the activation of memory CD4 T cells (58). Six hundred seventy-two mutations in 45 different CCA genes were also detected by Ali Shah et al. (59). With three long short-term models, gated recurrent units, and bidirectional LSTM algorithms based on RNN, the deep learning model was established with a sensitivity of 100%, specificity of 98%, and accuracy of 99%. Liao et al. (60) screened out 166 CCA disease signature genes by RF algorithms and built an ANN prediction model for CCA, with an AUC of 0.980. Genomic profiles were used to identify the gene alteration of HCC, CCA, and cHCC-CCA. Murugesan et al. (61) built an RF model to classify cHCC-CCA by integrating genomic-derived data. TP53, TERT, and PTEN were the most common in gene alterations. Combined with the DNA methylation data from public data and in-house patients, RF, SVM, and NN were developed. Further improving by anomaly detection, the accuracies were continually enhanced, and the best balance was reached by NN concerning accuracy and the number of samples (62). With the single-cell RNA-sequencing datasets, Swain et al. (63) used KNN and SNN to cluster the cell group. Moreover, UMAP and t-SNE identified the top 2 components to classify HCC and ICC. Further analysis indicated the interaction between smooth muscle cells and epithelial cells and cell adhesion pathway alteration in ICC. Transcriptomic data from public datasets were used to identify the classification between ICC and ECC. Differentially expressed genes in EMT, DNA repair, and the EGFR pathway were identified. KNN, SVM, and fast unified RF were used to build the prediction model (64). The translational proteomic approach was also used to discover specific CCA biomarkers. Seven candidate proteins and four significantly increased proteins proven by enzyme-linked immunosorbent assays with SVM showed strong predictive performances for CCA, with an AUC of 0.96 (28).

Imaging technology plays an important part in the diagnosis of CCA. Current technology includes ultrasound, CT, MRI, and 18F-FDG PET/CT. Due to similar invasive features of hepatic alveolar echinococcosis with malignancy, Wa et al. (65) utilized ultrasound and CEUS features with LASSO regression to establish the US scoring system for differentiation.

The first study with the application of the AI algorithm in CCA CT images was in 2006 (66). HCC is a liver tumor due to the abnormal proliferation of parenchyma, whereas dysregulation of epithelioid cells of the biliary tract results in ICC. ANN is designed based on the 24 CT image features and 9 clinical findings. The AUC was 0.961 and the radiologist’s performance with the ANN model significantly improved (*p* < 0.02). With multiphase CT scan images, SVM was utilized by Ponnoprat et al. to develop a method and achieve an accuracy of 88% in the classification of liver cancer (67). Portal venous phase images from CT scans analyzed by the modified Inception v3 network algorithms obtained an accuracy of 96% and sensitivity of 94% in ICC classification (68). Combining CT images with the tumor markers, the CNN model was developed to categorize ICC from HCC, achieving better accuracy and specificity than the radiologist (43).

Different from the previous study concerning gene expression profiles, several phases of the CE-CT were enrolled in the study to investigate the different radiological features in different CCA-related gene expression levels (58, 59). The IDH gene, which translated key enzymes in the tricarboxylic acid cycle, frequently mutated and participated in the carcinogenesis of ICC. Venous phase images showed great accuracy of 86.3% and specificity of 88.5% in predicting the IDH mutations with the SVM algorithm (69).

MRI has become the better choice for the staging and differentiation of CCA because of the high sensitivity of the infiltration and high resolution. Shen et al. (70) developed a nomogram based on LASSO and RF to identify ICC among intrahepatic lithiasis patients, with an accuracy of 82.6%. Hu et al. (71) made use of a tree-based pipeline optimization tool. After manual segmentation of the tumors, different thresholds of feature selections and classifiers were evaluated. The best combinations were established with an accuracy of 73%–75%. To differentiate the cHCC-CCA, the LASSO model, composed of clinical features (tumor size, age, etc.) and MRI images, was built with an AUC of 0.863 and specificity of 91.8% (72). CNN with multiphasic MRI features achieved a specificity of 98% and AUC of 0.992 in classifying six hepatic tumors. Further study about the radiologic imaging features for each hepatic tumor reached a PPV of 83.3% in differentiating the ICC from liver lesions (41, 42). The strided feature fusion residual network, a deep learning workflow composed of MFF, SRB, and CBAM, was also proposed to automatically utilize the MRI features to distinguish ICC, with an AUC of 0.9680 (73). Compared with the deep learning workflow, Gd‐EOB‐DTPA-enhanced MRI features with RF, SGD, and SVM algorithms achieved an AUC of 0.90 in differentiating atypical intrahepatic mass-forming CCA from poorly differentiated HCC.

Radiomics is defined as the extraction and analysis of advanced quantitative features from medical images of ultrasound, CT, MRI, and 18F-FDG PET/CT. Radiomics features can be extracted after the region of interest is delineated. Several algorithms were used to establish the predictive model and had favorable value to predict CCA from infected focal liver lesions (74). Ren et al. (75) performed SVM based on ultrasonics to distinguish ICC from HCC, with an AUC of 0.936 and a sensitivity of 0.900. Also, the ultrasound-based radiomics with LASSO algorithm analysis achieved an AUC of 0.920 (76). Xu et al. (77) employed 45 models based on the combination of 5 selection methods and 9 classification methods to study radiomics in CE-CT. The RF algorithm combined with LDA achieved the highest AUC and accuracy (0.997 and 0.969, respectively) to differentiate between ICC and hepatic lymphoma. Combined CT radiomics features and clinical features can detect early malignant lesions and predict the tumor prognosis. Utilized by Mahmoudi et al. (19), four independent ML models were established and LR achieved the best AUC of 0.82. A better AUC of 0.855 was achieved by Xu et al. with LASSO and SVM algorithms to classify ICC with CT radiomics features (78). Moreover, SVM was conducted by Liu et al. to differentiate cHCC-CCA by using MRI radiomics features, with an AUC of 0.81, while CT radiomics was of limited value (79). Meanwhile, LASSO and SVM may provide a better AUC of 0.91 and an accuracy of 89% with MRI radiomics to distinguish different pathological types of liver cancer, including cHCC-CCA, HCC, and ICC. The workflow for optimal radiomics classification toolbox, involving eight machine learning approaches, analyzed T2-weighted MRI radiomics to distinguish malignant and benign primary solid liver lesions, with an AUC of 0.78. Furthermore, the CSAM-Net model was established with better AUC values and accuracy than those of the conventional radiomics models (80).

Microscopic hyperspectral (HSI) pathological images of CCA are collected, typically with more pixel information than the traditional RGB images. Exploration was conducted using deep CNN and further RF algorithms to predict CCA on a pathological patch size of 299, with an accuracy of 88.2% (81). Optical coherence tomography is available for scanning the resection specimens to determine the resection margins intraoperatively. Trained with CNN, the sensitivity and specificity reached 94% and 93%, respectively (82). To identify the impact of AI algorithms in assisting pathologists to differentiate between HCC and CCA, CNN was trained to classify hematoxylin and eosin-stained whole-slide images, with an accuracy of 0.885. The algorithm improved the accuracy of the well-experienced pathologists (*p* = 0.045). Meanwhile, the accuracy of the algorithm had a great impact on the decision-making of the pathologists (83).

## AI and prognosis

Accurately predicting the prognosis of CCA is important for individual treatment strategy. For potentially curative cholangiocarcinoma, the long-term prognosis depends on various factors, including the location and stage of the primary lesion, surgery-associated complications, and treatment-related complications (1, 2, 84, 85). The main prognostic factors are margin status and lymph node involvement (86, 87). For advanced cholangiocarcinoma, the prognosis is mainly predicted by the M stage, primary lesion site, and elevated serum alkaline phosphatase levels (88). Chemotherapy and clinical trials may not significantly improve the prognosis (89).

**Table 2** summarized the application of AI in prognosis of cholangiocarcinoma. Clinical features and TNM stage are frequently used in predicting the survival and prognosis of CCA patients. Classification algorithms can easily identify the important factors concerning the prognosis. To detect the burden of the ICC and predict the prognosis, the CART algorithm was used to find out the best estimator. Lymph node metastasis and tumor size had a great effect on long-term prognosis (90). Also, the CART algorithm was used by Kaibori to make risk classification in ICC. Preoperative biomarkers, including CA 19-9, CRP, and CAR (ratio between CRP and albumin), were used for predicting OS and RFS (91). Tsilimigras et al. (92) divided the ICC into three phenotypes by tumor size, CA 19-9, and NLR. The classification tree algorithm assigned patients to different clusters and indicated different OS rates. Zhou et al. (93) established three RF models and developed an online tool, with an AUC of 0.7478. Serum albumin-to-fibrinogen ratio and CA 19-9 were used to develop the DeepSurv model to predict the prognosis of ECC and guide individualized postoperative chemotherapy as shown in the study by Wang et al. (87). The prognostic scoring system of ICC was constructed based on XGBoost, RF, and GBDT to evaluate the prognosis by biomarkers, with a C-index of 0.693 (94). The psoas muscle index, defined as the area of the psoas muscle at the L3 vertebra level divided by the squared body height, combined with the features of tumor burden and hepatic reserve in ANN, with an AUC of 0.89 in the 1-year survival prediction in ICC, was significantly higher than the Fudan score (95, 96). Clinical risk factors and CT radiomics, selected by the LASSO algorithm, were also used to build a nomogram to preoperatively predict the prognosis of patients with ICC, with an AUC of 0.783 in a 3-year OS (97). To identify patients who will benefit the most from surgery, Tsilimigras et al. (98) further utilized preoperative estimated tumor number and size, albumin–bilirubin (ALBI) grade, and preoperative lymph node by the CART algorithm to generate four different groups, achieving different R0 resection rates, microvascular invasion rates, 5-year OS, and 5-year DFS. Clinical features and serum biomarkers were conveniently obtained and trained with the algorithms. Tools established by those features can be easily found online, but they may not provide a more accurate prediction of prognosis.

**Table 2:** Application of AI in the prognosis of cholangiocarcinoma.

Reference	Sample size	Data source	Algorithms	Aim	Best result
Zhou et al.	4,398 + 504 cases	Clinical features	RF	Predict the short-term prognosis of ICC	C-index 0.73, AUC 0.7478
Qin et al.	641 cases	CE-CT, clinical features, and molecular features	LASSO	Predict early recurrence after curative resection of PHC	AUC 0.883, accuracy 0.826, sensitivity 0.810
Ji et al.	649 + 401 cases	Clinical features and surgery information	GBM	Predict prognosis after liver resection of ICC	C-statistic 0.751
Alaimo et al.	536 cases	Clinicopathologic characteristics	RF	Predict the recurrence of ICC	AUC 0.904
Ruan et al.	266 cases	Proteome and transcriptome datasets	LASSO	Predict metastasis and risk stratification of ICC	Accuracy 97.1%, AUC 0.958
Zhang et al.	98 cases	AP and PVP MRI	LASSO	Predict PD-1/PD-L1 expression and outcome in ICC	AUC 0.897, C-index 0.721
Wang et al.	169 cases	Clinicopathologic characteristics	DeepSurv, LASSO, RF	Predict prognosis and guide postoperative chemotherapy for distal cholangiocarcinoma	C-index 0.746, AUC 0.823
Alaimo et al.	600 cases	Clinicopathologic characteristics	RF	Assess optimal margin width in hepatectomy and long-term outcomes of ICC	AUC 0.81
Jolissaint et al.	138 cases	CT	RF	Predict early liver recurrence after resection of ICC	AUC 0.84, sensitivity 0.91, specificity 0.57, PPV 0.44, and NPV 0.94
Song et al.	311 cases	CT and clinical features	LightGBM	Predict early recurrence risk after curative resection of ICC	AUC 0.974
Li et al.	1,390 cases	Clinical features	XGBoost, RF, and GBDT	Prognostic scoring system of ICC	C-index 0.693
Bo et al.	127 patients	AP and PVP CE-CT	LR, RF, NN, Bayes, SVM, LightGBM, and XGBoost	Predict early recurrence of ICC after curative resection	AUC 0.89
Muller et al.	417 patients	Clinical features	ANN	Survival prediction in ICC	AUC 0.89
Tang et al.	101 cases	CT radiomics and clinical features	LASSO	Prognostic nomogram of ICC	AUC 0.783
Tsilimigras et al.	1,146 cases	Demographic and clinicopathologic data	CART	Identify the benefit associated with resection for ICC	–
Bagante et al.	1,116 cases	Clinical features	CART	Define the prognostic ICC groups after resection	–
Kaibori et al.	225 cases	Preoperative blood test biomarkers	CART	Serum markers and risk classification of ICC	–
Ibragimov et al.	122 cases	CT images and delivered dose plans	CNN	Recognition of consistent dose patterns and generation of toxicity risk maps of chemotherapy	Accuracy 0.73
Xie et al.	127 cases	H&E-stained whole-slide images	mRMR, LDA, QDA, and DT	Predict survival in ICC	AUC 0.74 ± 0.06, accuracy 0.70 ± 0.10, specificity 0.74 ± 0.10, sensitivity of 0.73 ± 0.11
Jeong et al.	1,421 cases	Serum biomarkers and clinicopathological features	TensorFlow DL algorithm	Latent ICC susceptible to adjuvant treatment risk after resection	AUC 0.78
Ibragimov et al.	125 cases	Treatment characteristics, CT image, and SBRT treatment plans	CNN, SVM, RF, and NN	Predict individualized hepatobiliary toxicity after liver SBRT	AUC 0.85
Plachouris et al.	19 cases	SPECT, CT scans, and clinical target volume	Generative adversarial network	Predict biodistribution of 90Y microspheres in liver radioembolization	–
Liang et al.	139 cases	AP contrast-enhanced MRI	LASSO	Predict early recurrence in ICC	AUC 0.90
Bagante et al.	649 genes	Whole-exome sequencing	ANN	Survival analysis of cholangiocarcinoma	C-index 0.71
Xu et al.	265 cases	CE-CT	DenseNet	Predict posthepatectomy liver failure after hemihepatectomy	Accuracy 84.15%, AUC 0.7927
Shao et al.	288 cases	Clinical features	ANN	Predict early occlusion of bilateral plastic stent placement	AUC 0.9648

CE-CT, contrast-enhanced computed tomography; MRI, magnetic resonance imaging; T1WI, T1-weighted imaging; T2WI, T2-weighted imaging; DWI, diffusion-weighted imaging; ADC, apparent diffusion coefficient; VEGF, vascular endothelial growth factor; MVD, microvessel density; AP, arterial phase; PVP, portal venous phase; 18F-FDG PET/CT, 18F-fluorodeoxyglucose positron emission tomography/computed tomography; CA 19-9, carbohydrate antigen 19-9; NLR, neutrophil-to-lymphocyte ratio; SBRT, stereotactic body radiotherapy; DL, deep learning; LASSO, least absolute shrinkage and selection operator; RF, random forest; SVM, support vector machine; ANN, artificial neural network; MFF, multilayer feature fusion module; SRB, stationary residual block; CRAM, convolutional block attention module; LR, logistic regression; SGD, stochastic gradient descent; NN, neural networks; t-SNE, t-distributed stochastic neighbor embedding; ADB, AdaBoost; SGB, stochastic gradient boosting; KNN, K-nearest neighbors; CNN, convolutional neural network; MLP, multilayer perception; DT, decision tree; LSTM, long short-term model, GRUs, gated recurrent units; SNN, shared nearest neighbor; UMAP, uniform manifold approximation and projection; XGBoost, extreme gradient boosting; mRMR, maximum relevance minimum redundancy; GBM, gradient boosting machine; CART, classification and regression tree; LDA, linear discriminant analysis; QDA, quadratic discriminant analysis; ICC, intrahepatic cholangiocarcinoma; ECC, extrahepatic cholangiocarcinoma; HCC, hepatocellular carcinoma; PHC, perihilar cholangiocarcinoma; AUC, area under the curve; NPV, negative predictive value; PPV, positive predictive value.

Molecular alterations of cancer cells were also useful as the prevalence of sequencing. Ruan et al. (99) combined LASSO and Cox regression to establish the final risk scoring of 21 gene-pair signatures to predict the prognosis, with an AUC of 0.88. Angiogenesis is a factor of rapid development and metastasis of malignancy, including CCA. Liu et al. (100) developed and validated a model based on several ML algorithms to predict vascular endothelial growth factor (VEGF) expression and microvessel density of ECC, with an AUC of 0.912. For further classifications and survival analysis of hepatobiliary cancers, ANN algorithms with whole-exome sequencing in the TCGA database revealed that IDH or METH-2 molecular subtypes of CCA may have a worse prognosis, with a C-index of 0.71 (90). Target therapy toward the PD-1/PD-L1 pathway has shown capability in improving CCA patients’ survival. The expression status of PD-1/PD-L1 was commonly assessed by immunohistochemical staining. Zhang et al. (101) first selected radiomics features from AP and PVP MR images to construct PD-1 and PD-L1 predictive models based on LASSO, with an AUC of 0.897. Xie et al. (102) focused on the tumor microenvironment of ICC and explored the lymphocyte number and segment features in the H&E-stained whole-slide images. After feature selection by the mRMR algorithm, three algorithms, namely, LDA, QDA, and DT, were selected to predict the survival of ICC, achieving the best AUC of 0.74 ± 0.06 by DT. Molecular subtyping of CCA with different prognoses now can be identified by several methods, which also provide patients with precision therapy options. However, such methods involve high costs.

A treatment strategy should be determined by surgeons to improve the long-term outcomes of CCA. For operable CCA, Alaimo et al. (103) set up an optimal policy tree model based on the RF algorithm to help surgeons determine margin width in hepatectomy, with an AUC of 0.81. Liver failure after hemihepatectomy for liver malignancy is the leading fatal reason for postoperative complications. For preoperative prediction, a dense neural network block was used to detect the radical information from CE-CT, with an accuracy of 84.15% (104). For inoperable hilar CCA, bilateral plastic stent placement is commonly used for maintaining biliary drainage. To predict the early occlusion of bilateral plastic stent placement, the ANN model analyzed the cancer stage and the Bismuth stage, achieving a larger AUC than the logistic regression model (0.9648 vs. 0.8763) (105). Radiation therapy and radioembolization with isotopes are available adjuvant therapies for CCA. Ibragimov et al. (106) combined CT images and delivered dose plans with the CNN algorithm to detect the specific anatomical regions with high hepatobiliary toxicity risk after radiation therapy. Consequently, the individual-delivered dose was determined with an accuracy of 0.73. Furthermore, the authors used the CNN + SVM+ RF + FcNN algorithms to predict the potential radiation toxicity of healthy organs and reach an AUC of 0.85 (34). Radioisotope 90Y microspheres in radioembolization were used in CCA treatment. The biodistribution of the isotopes used to be predicted with difficulty, but generative adversarial network algorithms with PET/CT scans and dose volume input have accurately predicted the biodistribution and are suitable for personalized pretreatment planning (107). AI now can provide better accuracy and simulation of the determination of the treatment strategy.

## Recurrence

Although surgery may offer the potential cure for localized and resectable CCA, the prognosis still remains low, with a 5-year survival of 25%–35%, and 50%–70% of patients experience recurrence (2). The most common type of recurrence for eCCA is local recurrence. Distant metastasis of cholangiocarcinoma is relatively common in hilar cholangiocarcinoma. A study showed that 41% of patients with hilar cholangiocarcinoma experienced initial recurrence involving distant areas (108). Other studies have reported that 60% of patients experienced distant metastasis after complete (R0) resection of the hilar cholangiocarcinoma under a microscope (109). On the other hand, the types of iCCA recurrence are intrahepatic recurrence, lymph node recurrence, and distant extrahepatic recurrence (usually in the peritoneum) (110). Consequently, identifying patients who may experience recurrence is important. Alaimo et al. (111) used 14 clinical features to conduct three ML algorithms. The RF algorithm shows better achievement than SVM and LR, with an AUC of 0.904 in the training set. Ji et al. (112) analyzed age, TMN stage, histological type, and surgery strategy to yield the GBM model to predict the prognosis of the ICC after liver resection. The model had improved prognostic discrimination (C-statistic, 0.723) compared with the AJCC staging system and MEGNA prognostic score system developed by Raoof et al. (86). Moreover, Jolissaint et al. (113) constructed the predictive model based on RF classification by the four tumor features and future liver remnant in CT, with an AUC of 0.84. Combining 15 radiomic features with 3 clinical features, Song et al. (18) constructed the LightGBM model with an AUC of 0.974.

Radiology also provides a methodological approach to predict the recurrence of cholangiocarcinoma. Qin et al. (114) combined clinical and molecular features with radiomic features from the CE-CT to make a model with good discrimination and sensitivity (AUC of 0.883 and accuracy of 0.826), which was superior to the 8th AJCC, MSKCC, and Gazzaniga staging systems in predicting the early recurrence of patients after curative resection in PHC (115–117). This model provided a comprehensive analysis of recurrence although some features may not be a high risk. To predict the early recurrence of ICC, Bo et al. (118) developed ML radiomics models with seven algorithms, with a mean AUC of 0.87 ± 0.02, and RF, NN, and SVM achieved the best performance, with an AUC of 0.89. With the same purpose, the arterial-phase image features of contrast-enhanced MRI, extracted by the LASSO algorithm, were used to establish a nomogram, with an AUC of 0.90, as shown by the study of Liang et al. (119).

## Metastasis

Several tumor cells in the bloodstream are considered early metastatic biomarkers for malignant tumors. To more efficiently detect circulating tumor cells, Piansaddhayanon et al. (120) established a large-scale microscopic imaging dataset of tumor and normal cells from organoid-derived cells of CCA. The model based on the deep NN algorithm provided a foundation for circulating tumor cell detection, with an AUC of 0.78, and may need further investigation.

Lymph node metastasis is an important factor for CCA prognosis. The 8th AJCC guideline demonstrated the stage of metastatic lymph nodes as a factor of poor prognosis. Recently, the radiomic models have gained great accuracy in predicting the lymph node status of CCA. Wang et al. (121) developed a model integrating SVM with clinical features to predict the lymph node stage of CCA patients, with an AUC of 0.870. To predict ICC lymph node metastasis preoperatively, serum CEA, CA 19-9, and lymphadenopathy on CT/MR image were enrolled in the RF training model. Compared with the nomogram based on logistic regression, the RF model has better sensitivity and accuracy (122). Xu et al. (123) investigated the image features from T1WI contrast-enhanced MRI. After extracting related features by the mRMR algorithm, the SVM model was built. Combined with clinical features, the nomogram was established with an AUC of 0.870. Tang et al. (124) focused on the differentiation degree and lymphatic node metastasis of ECC predicted by MRI radiomics. Four MRI sequence features and six clinical features were selected by five methods, including JMI, mRMR, SKB, and Wilcoxon. Then, 10 machine learning algorithms were conducted. The bagging classifier gained the best performance for differentiation degree, and XGB gained the best performance for lymphatic node metastasis, both significantly different from the other eight models (ADB, DT, Gaussian naive Bayesian, etc.). To preoperatively predict the microvascular invasion in ICC, several parametric MRI images were fused by the CNN algorithms. Gradient-weighted class activation mapping is used for visualizing the network and highlighting the important factors in estimation, with a sensitivity of 85.7% and accuracy of 86.8% (125). Consequently, lymph node metastasis nowadays can be predicted with great sensitivity and accuracy, while other characteristics including microvascular invasion are less studied.

Gene and protein expression profiling acts as a potential approach to malignant diagnosis and prognosis, but not in the metastasis prediction due to the batch effects. Therefore, genome-wide integrative proteome and transcriptome analysis was necessary. Ruan et al. (99) assembled models by using their own proteome and transcriptome datasets, validated by the TCGA dataset and the Ahn dataset. Developed from the k-TSP method, the authors employed a weighted voting procedure for the gene pairs to finally predict the metastasis possibilities. The EMLI algorithm achieved an AUC of 0.958 and an accuracy of 97.1% in ICC patients. However, detection of the proteome and transcriptome still involves a high cost and may not be widely accepted in medical practice.

## AI and other aspects

Fibroblast growth factor receptors bind fibroblast growth factor and mediate cellular functions. With a dataset of 2,356 chemical compounds, Charan et al. utilized four ML algorithms (SVM, RF, k-NN, and ANN) to screen potential FGFR1 inhibitors for various cancers, including CCA (126). Furthermore, to predict the efficacy of anticancer drugs in individual patients, Gerdes et al. (127) used the proteomics and phosphoproteomics data from various cell lines to train and verify drug ranking using the ML algorithm, which was based on the DL, NNET, BGLM, RF, PLS, PCR, SVM, and cubist ML models. This algorithm has achieved quite a low error, with a mean-squared error <0.1. In CCA patients, inhibitors of histone deacetylase and the PI3K pathway were selected as high-ranking therapies (128). The TensorFlow deep learning algorithm was used to investigate the adjuvant treatment risk for ICC patients. A total of 8 features including tumor burden, CA 19-9, and CEA were selected as significant prognostic factors with the TensorFlow deep learning algorithm, reaching an AUC of 0.78, which was higher than the AJCC stage prediction (129).

The latent Dirichlet allocation algorithm, a machine learning method to identify research topics, was used to summarize scientific publications in the field of CCA. The results demonstrated that survival and differential diagnosis were the highly concerned research topics of CCA, which were in accordance with the conclusion of this review. Although several studies are concerned about microRNA expression, high-quality clinical trials and basic studies are still urgently needed (130).

## Discussion

Several studies have investigated the application of AI on malignancy in several aspects of diagnosis, prognosis, recurrence, and metastasis with some extent of automation. Moreover, it is obvious that AI is already better than people in some areas as a consequence of technological development. However, not a single algorithm can solve all problems as different algorithms are suitable for different scenarios. Artificial intelligence algorithms are difficult to completely replace human decision-making and judgment.

Although AI algorithms have many involvements and gain great results in the daily medical work of CCA, many challenges still remain (30, 40, 131, 132). AI requires a large amount of data for training and optimization, but some data are difficult to obtain because the equipment needed may be quite expensive so they may not be available in a single center trial. Moreover, many medical slides may contain millions of pixels and are too large to be fed into the algorithms. Once the whole image is split into small slides to input, some potential trends may be ignored by the algorithms. On the other hand, some medical data lack labels necessary for supervised learning, and the quality of input data may be varied due to the different experiences of the experts. This may significantly affect the accuracy of the model, and handling large datasets becomes difficult.

The interpretability of the algorithms, the degree to which machine learning algorithms can be understood by humans, is still the biggest challenge for their wide usage in the field of medicine. There are several ethical challenges related to AI in the medical field. The most obvious part is distrusting the accuracy of AI prediction as the replicability of specific AI is hard to achieve without publicly released data. Furthermore, the continual learning capability of AI also raises challenges in its regulation. In 2019, the FDA proposed a framework to evaluate AI products from premarket performance to postmarket performance (https://www.fda.gov/medical-device). In addition, it is unclear whether developers or doctors are to be blamed when AI models make mistakes (133).

Nowadays, three approaches have attempted to explain the interpretability of AI models: 1) surrogate models, which use a simple and interpretable model to approximate the original model. When the accuracy is close enough, a surrogate model can be used to explain the original “black-box” model; 2) intrinsic interpretability, which shows the workflow of the algorithms and compares with the mind of humans; and 3) data visualization, which helps us to quickly and comprehensively understand the characteristics of data distribution, thereby assisting us in choosing the most reasonable model to approximate the optimal solution that the problem can achieve. These three methods may provide us with the way to test the safety and efficacy of the AI algorithms before application in the medical field and avoid patient harm (134, 135).

With further development of large databases and improvement of computing power, AI will be trained with clinical features based on multicenter and multiomics data to achieve better clinical decision-making capability. Despite the challenges of AI, it will eventually be integrated into the diagnosis and treatment of cholangiocarcinoma due to its great advantage in clinical practice. Apart from radiology and serum biomarkers, cell-free tumor DNA in whole genome sequencing can be studied by AI to identify early cholangiocarcinoma and predict patient outcomes (136). It can be assumed that early detection of cholangiocarcinoma may be achieved with the corporative training of physicians and the use of AI. AI also creates the need for new capabilities in data processing and machine learning.

## Conclusions

We concluded the applications of AI on CCA. Early detection can be achieved by the combination of medical data and AI. Moreover, AI assists physicians in making accurate diagnoses and proper treatment options. Several studies focused on the diagnosis of and survival from cholangiocarcinoma, and high-quality clinical trials are still urgently needed for cholangiocarcinoma patients. Despite some limitations of current AI applications, AI will still significantly improve many aspects of cholangiocarcinoma in the medical field.

## Author contributions

JH: Conceptualization, Data curation, Formal Analysis, Investigation, Methodology, Project administration, Resources, Software, Visualization, Writing – original draft. YQ: Conceptualization, Data curation, Formal Analysis, Investigation, Methodology, Project administration, Resources, Software, Visualization, Writing – original draft. XB: Conceptualization, Data curation, Formal Analysis, Investigation, Methodology, Project administration, Resources, Software, Visualization, Writing – original draft. XH: Conceptualization, Funding acquisition, Validation, Writing – review & editing.

## Glossary

**Table:**

ERCP	endoscopic retrograde cholangiopancreatography
CE-CT	contrast-enhanced computed tomography
MRI	magnetic resonance imaging
T1WI	T1-weighted imaging
T2WI	T2-weighted imaging
DWI	diffusion-weighted imaging
CEUS	contrast-enhanced ultrasonography
ADC	apparent diffusion coefficient
VEGF	vascular endothelial growth factor
MVD	microvessel density
AP	arterial phase
PVP	portal venous phase
18F-FDG PET/CT	18F-fluorodeoxyglucose positron emission tomography/computed tomography
CA 19-9	carbohydrate antigen 19-9
NLR	neutrophil-to-lymphocyte ratio
SBRT	stereotactic body radiotherapy
qRT-PCR	quantitative real-time PCR
DL	deep learning
LASSO	least absolute shrinkage and selection operator
RF	random forest
SVM	support vector machine
ANN	artificial neural network
MFF	multilayer feature fusion module
SRB	stationary residual block
CRAM	convolutional block attention module
LR	logistic regression
SGD	stochastic gradient descent
NN	neural network
t-SNE	t-distributed stochastic neighbor embedding
ADB	AdaBoost
SGB	stochastic gradient boosting
KNN	K-nearest neighbors
CNN	convolutional neural network
MLP	multilayer perception
DT	decision tree
LSTM	long short-term model
GRUs	gated recurrent units
SNN	shared nearest neighbor
UMAP	uniform manifold approximation and projection
XGBoost	extreme gradient boosting
mRMR	maximum relevance minimum redundancy
GBM	gradient boosting machine
CART	classification and regression tree
LDA	linear discriminant analysis
QDA	quadratic discriminant analysis
PCA	principal component analysis
SVD	singular value decomposition
RNN	recurrent neural network
JMI	joint mutual information
SKB	select K best-using analysis of variance
PCR	principal components regression
BGLM	Bayesian estimation in generalized linear model
ICC	intrahepatic cholangiocarcinoma
ECC	extrahepatic cholangiocarcinoma
HCC	hepatocellular carcinoma
PHC	perihilar cholangiocarcinoma
cHCC-CCA	combined hepatocellular cholangiocarcinoma
AURPC	area under the receiver operating characteristics curve
AUC	area under the curve
NPV	negative predictive value
PPV	positive predictive value.
